# Strengthening the mechanical characteristics and cathodic delamination resistance of fiber-reinforced polymer through chemical surface modification of glass fibers

**DOI:** 10.1038/s41598-023-40555-1

**Published:** 2023-08-17

**Authors:** M. Shariatmadar, S. Feizollahi, P. Gholamhosseini, Z. Abdorrezaee, S. Ghorbanzadeh, F. S. Hosseini, F. Azad Shahraki, M. Mahdavian

**Affiliations:** 1 Department of Surface Coatings and Corrosion, Institute for Color Science and Technology, Tehran, Iran; 2 Qazvin Water and Wastewater Company, Qazvin, Iran

**Keywords:** Materials science, Structural materials, Composites, Glasses, Mechanical properties, Metals and alloys

## Abstract

This work aims to scrutinize the effect of the silanization of glass fibers (GF) on the mechanical properties and cathodic disbonding resistance of an epoxy composite coating. Successful silanization was approved based on different characterization techniques, including Fourier transform infrared spectra, field emission-scanning electron microscopy (FE-SEM), energy-dispersive X-ray spectroscopy, and thermogravimetric analysis. Tensile strength measurement exhibited a significant effect of silanization on the mechanical performance of the fiber-reinforced polymer (FRP). FE-SEM cross-sectional images illustrated improved interfacial bonding between the epoxy matrix and GF upon silanization. Pull-off measurements revealed improved wet adhesion strength of the FRP to the mild steel surface after exposure to the salt spray chamber when the GF were silanized. In addition, silanization revealed enhanced resistance to cathodic delamination (CD). Electrochemical impedance spectroscopy and electrochemical noise assessments proved silanization's significant influence on the FRP's CD resistance.

## Introduction

Epoxy polymers are broadly utilized as popular corrosion protection coatings in different applications due to their many exceptional properties, including excellent chemical resistance, toughness, shrinkage resistance, and adhesion^1–4^. Epoxy coatings act as an efficient barrier to water/corrosive species transfer to the metal substrates and increase their service life by reducing the corrosion rate from severe corrosive media. Polymer coatings can significantly decrease metallic structure corrosion via three dominant mechanisms: barrier, inhibition, and sacrificial^5–9^.

Generally, the organic coatings are relatively penetrable to water, oxygen, and corrosive species. Hence, after being exposed to corrosive electrolytes, the coatings undergo a degradation process, usually by forming defects such as cracking and delamination^10,11^. This also leads to a severe decrement in the barrier performance of the coating, leading to the penetration of more water and corrosive species into the interface of coating and substrate and acceleration of the metal corrosion rate. Loss of adhesion and delamination of the coating expands the cathodic and anodic areas increasing the rate of electrochemical reactions.

Various factors, such as interfacial interactions between the substrate and the polymeric coating, influence the robustness of the coating in corrosive media^12^. Many efforts have been made to enhance the coatings' adhesion to metallic substrates because the loss of adhesion directly affects the protective behaviors of polymeric coatings^13,14^.

Studies have shown that various additives or anticorrosion pigments increase the barrier functionality and protection features of polymeric coatings^15^. Recently, a variety of micro/nano reinforcers in the polymer matrix have been used to produce efficient composite coatings with higher mechanical strength, corrosion protection, and thermal and chemical stability^16–19^. The utilized nanoparticles in literature can be divided by their dimensions: (I) 0-dimensional including silica nanoparticles^20^ and carbon quantum dots^21^, (II) 1-dimensional including nanofibers and nanotubes such as carbon fiber^22^ and carbon nanotubes (CNT)^23,24^, (III) 2-dimensional including nanoplates and nanosheets such as graphene-based materials^25–27^, molybdenum disulfide^28^, layered double hydroxides (LDHs)^29,30^, and (IV) 3-dimensional including organic metal frameworks (MOF)^31^ and zeolites^32^.

Glass fibers (GF) are probably the most widely used reinforcing fillers in polymer composites. These composites are excellent and have low density, robust thermal and chemical stability, high rigidity and strength, and superior corrosion resistance^33^. Although these properties, GF are also prone to a variety of defects such as cracking, delamination, and failure during loading. Many of the locations that cause these defects result from poor bonding between GF and matrix, which can affect the material's mechanical strength^34,35^. Thus, solving this limitation improves the imperfect surface adhesion between the fibers and the polymer matrix and prepares a multifunctional composite with robust mechanical and protective features. Researchers around the world reported that the most important practical solutions to overcome this limitation are: (1) using coupling agents in the polymeric matrix^36,37^ and (2) surface treatment of fibers^38^. Various surface modification techniques have been utilized on GF to improve their interfacial interactions with polymeric matrixes. Alkali treatment, acetylation, plasma treatment, and grafting are common surface modification approaches for GF^39^.

Zhao et al.^37^ used polyhedral oligomeric silsesquioxanes as an efficacious coupling agent for surface modification of conventional carbon fibers. Results showed that the interfacial bonding solidity of the composites increased by enhancing the wettability and chemical linkage. In recent research, Wang et al.^40^ used silane coupling agents to treat nano-SiO_2_ in a cellulose matrix, and the results indicated the KH-550 as an optimum modifier. Also, Feng et al.^41^ investigated the consequence of alkaline and organosilane modifications on the mechanical features of polypropylene composites improved with kenaf fibers and pineapple leaves. The results showed that mechanical properties increased with chemical treatments.

The aim of this study is the surface modification of GF with (3-aminopropyl) triethoxysilane (APTES) and utilize it in an epoxy matrix as a robust FRP. The mechanical properties, cathodic delamination resistance, and adhesion of the modified and unmodified FRP were investigated in this work. This study is novel from the previous publications as it studies for the first time the consequence of silanization of GF on the cathodic resistance of the final FRP coating system. The cathodic disbonding resistance measurements were accompanied by two complementary electrochemical techniques: electrochemical noise (EN) and electrochemical impedance spectroscopy (EIS).

## Experimental

### Materials

APTES was acquired from Merck Millipore (Germany). GF (GF/chopped strand mat) were obtained from Tamasha company (Iran). Zinc phosphate (ZP10) was provided by Heubach (Germany). Mild steel (ST12, Foolad Mobarakeh, Iran) was used as the substrate, and industrial epoxy resin (Epiran 01 and Epiran 06) and hardener (polyamide/SH615 and polyamine/F205, respectively) were employed for the fabrication of primer and top coatings, respectively.

### Surface modification of GF

**Figure 1:**
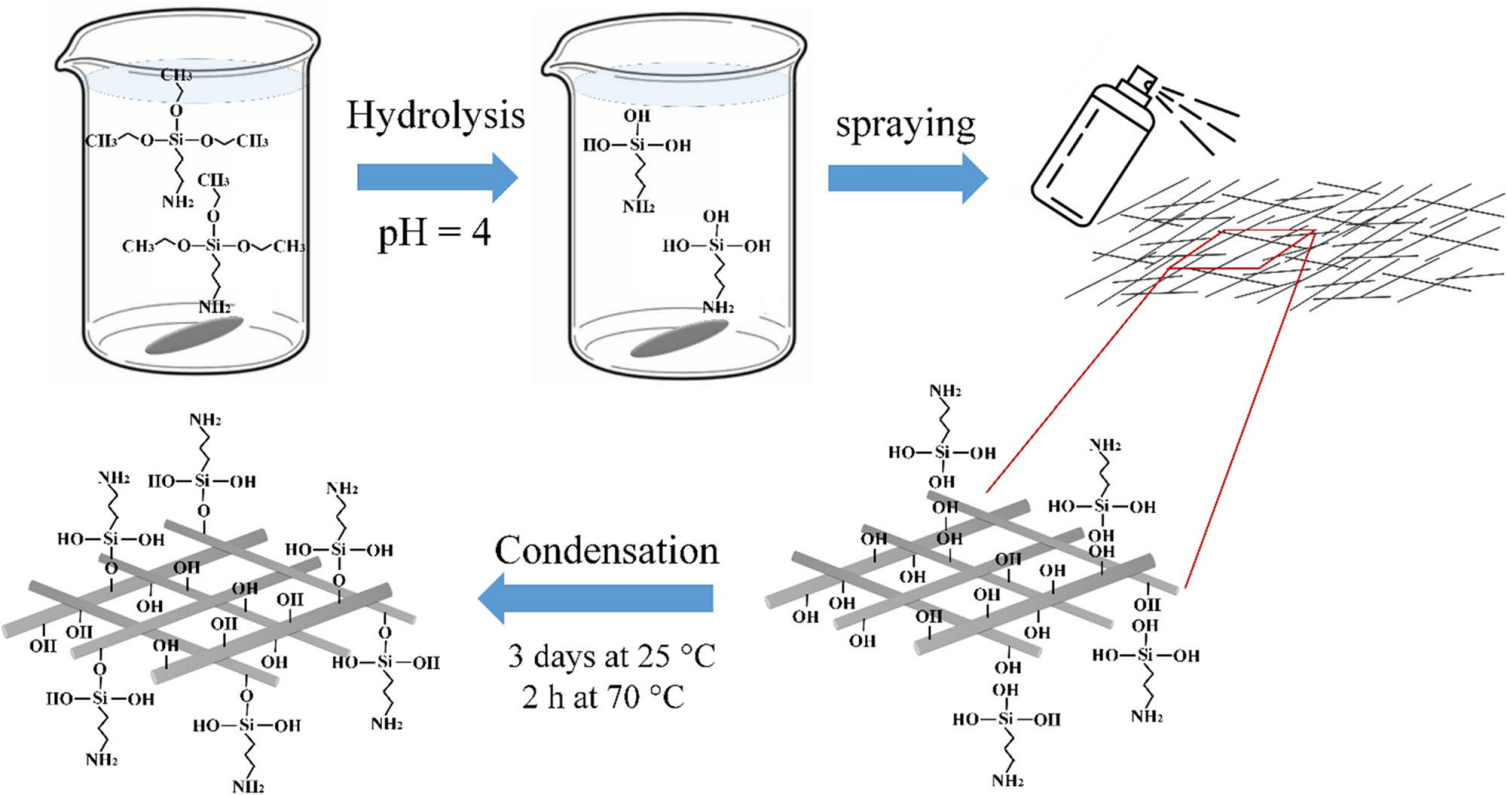
The schematic modification of GFs with APTES.

A silane solution was made by stirring 20 g of APTES into a mixture of 70 g of ethanol and 30 g of deionized water. The pH was brought to 4 for the hydrolysis of APTES by adding acetic acid and stirring for 4 h at 25 °C. Afterward, the silane solution was sprayed onto the GF, and the modified fibers denoted as MGF, were maintained at 25 °C for 3 days and 2 h at 75 °C to speed up the completion of the condensation reactions. The illustrative procedure of the surface modification is depicted in Fig. 1.

### Characterization of GF and MGF

Fourier transform infrared spectra (FTIR) of GF and MGF samples were obtained using utilizing a Perkin Elmer spectrometer over the wavenumber span of 400–4000 cm^−1^. Field emission-scanning electron microscopy (FE-SEM) was performed utilizing a TESCAN instrument to assess the surface morphology and structure of the specimens. The thermal behavior of GF was investigated by thermogravimetric analysis (TGA) employing a Mettler Toledo instrument. Measurements were conducted at the temperature span of 20–600 °C with 10 °C min^−1^ heating rate.

### Water contact angle test

A contact angle instrument (OCA 15 plus) was utilized to evaluate the wettability of the GF and MGF. In this experiment, a precisely measured droplet of deionized water (4 μL) was placed onto the compact GF and MGF under controlled conditions of 25 ± 2 °C and 30 ± 5% humidity. After a 10-s, the water droplet's appearance was captured using a Canon digital camera.

### Preparation of FRP samples

The FRP samples were prepared for cathodic delamination and EIS measurements test. To this end, the carbon steel panels were polished with sandpaper, washed with xylene, and dried. Afterward, the plates were coated with an epoxy primer coating containing 10 wt% ZP10 anticorrosive pigment, 60 wt% epoxy resin (Epiran 01), 30 wt% hardener (polyamide). The coated panels were cured at 25 °C for 7 days and post-cured in an oven at 60 °C for 2 h. A sheet of GF was fixed on the coated plate, and the mixture of 66.6 wt% epoxy resin (Epiran 06) and 33.4 wt% hardener (polyamine) was applied onto the GF. Finally, the FRP specimens were cured at 25 °C for 7 days and post-cured in the oven at 60 °C for 2 h. A similar procedure was followed for MGF, and the final composite was denoted as MFRP.

### Mechanical and compatibility tests

To evaluate the tensile strength and compatibility of GF and MGF in epoxy resin, the 6.5 mm × 12 mm × 100 mm specimen was prepared using silicone molds, following the same resin and curing conditions mentioned in "Water contact angle test". The tensile strength of specimens was assessed by SANTAM (STM-5, EQSC1-22) instrument at the strain of 5 mm min^−1^. The fracture area of the specimens was examined by SEM and EDS analysis utilizing the TESCAN instrument.

### Cathodic disbonding and electrochemical measurements

The cathodic delamination measurements were taken from the specimens immersed in saline solution (3.5 wt% NaCl). A pit with 5 mm diameter was excavated at the center of the prepared FRP and MFRP samples (2 cm × 2 cm), and a potential of − 1.5 V vs. saturated calomel electrode (SCE) was imposed for 7 days in the 3.5% NaCl solution utilizing platin wire as the counter electrode.

The EIS measurements were carried out from the sample before and after the cathodic delamination test using a three-electrode setup, including SCE as the reference electrode, FRP and MFRP coated specimens as working electrodes, and platin as the counter electrode. The EIS measurement was implemented at open-circuit potential (OCP) in the frequency span of 10 kHz–10 mHz. The EIS data were fitted by ZSimpWin3.1 software.

The EN was implemented in a period of 800 s at 0.1 s intervals by connecting the two identical samples after 6 days of exposure to the cathodic delamination condition. The SCE was employed as the reference electrode. Wavelet transformation was utilized to remove the smooth crystal (DC trend) and to plot the relative amplitude of transients in the frequency domain.

### Adhesion strength measurement

The pull-off test was conducted according to ASTM D4541 on all specimens in dry (before exposure to salt spray) and wet (after 720 h subjection to salt spray) states. The salt spray condition was in accordance with the ASTM B117. Adhesion measurements were conducted utilizing a PosiTest pull-off adhesion tester (DeFelsko, USA) at 10 mm min^−1^ velocity.

## Results and discussion

### Characterization of silanized GF

**Figure 2:**
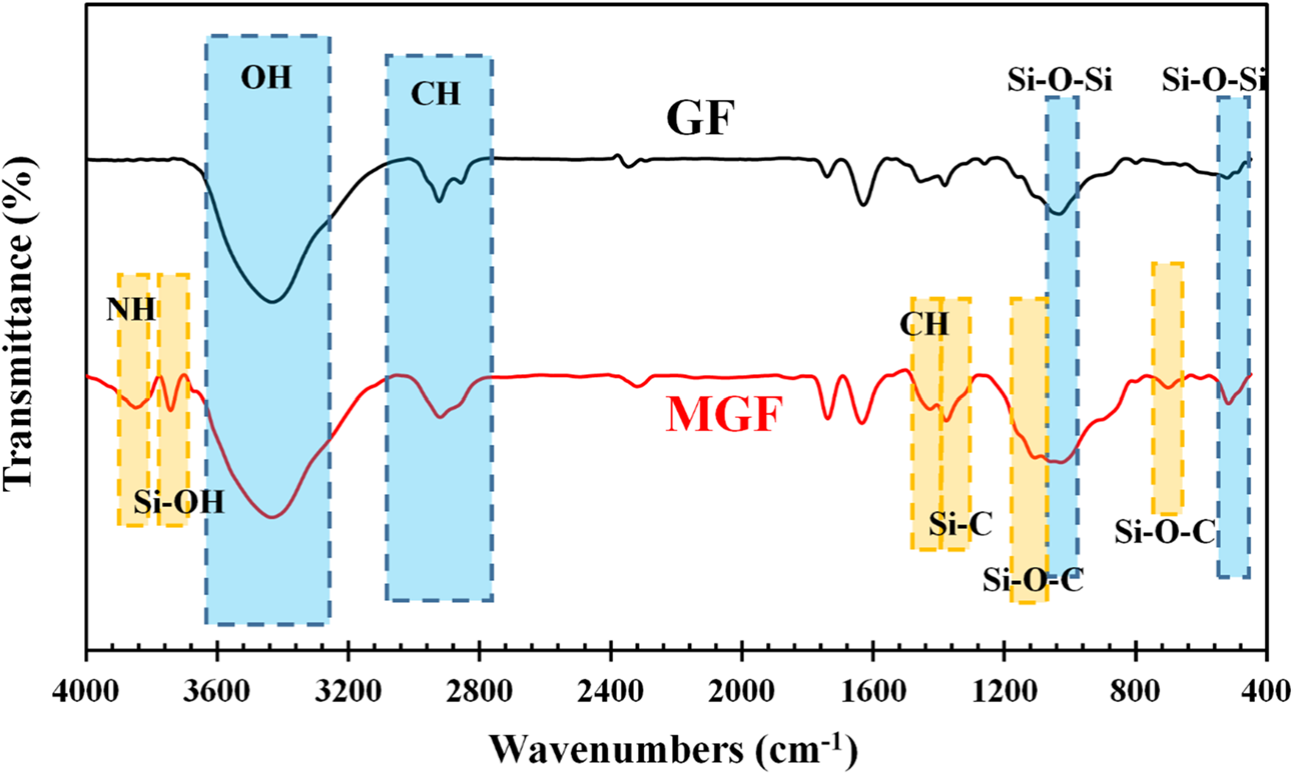
The ATR-FTIR spectra of GF and MGF.

Figure 2 illustrates the FTIR measurement results of modified and unmodified GFs. In the spectrum of neat GF, stretching vibrations of Si–O–Si can be observed at 1043 cm^−1^, while bending vibrations take place at 522 and 800 cm^−1^. The absorption bands at 800 and 3433 cm^−1^ could also be connected to Si–OH and hydroxyl group vibration, respectively. The absorption bands at 2850 and 2924 cm^−1^ are due to the stretching vibration of C–H in CH_2_ and CH_3_ groups. The bands at 1630 and 1741 cm^−1^ are analogous to the stretching vibration of C=C and C=O. Asymmetric and symmetric bending vibrations of C–H appeared at 1382 and 1455 cm^−1^^42–45^.

Compared to the neat GF, some new peaks appeared, and some peaks have been intensified for MGF. The bands at 702, 1040, and 1116 cm^−1^ are due to the Si–O–C vibrations, and the bands at around 1430 and 3850 are linked to the stretching vibration of C–H and N–H, respectively^46,47^. Moreover, the appearance of the peak at 3743 cm^−1^ was assigned to the unreacted silanol (Si–OH) group^48^. The magnitude of the band at 1378 cm^−1^ rises owing to the increment of Si–C and C–H groups^49–51^. Therefore, these results confirm the successful modification of GF with APTES.

**Figure 3:**
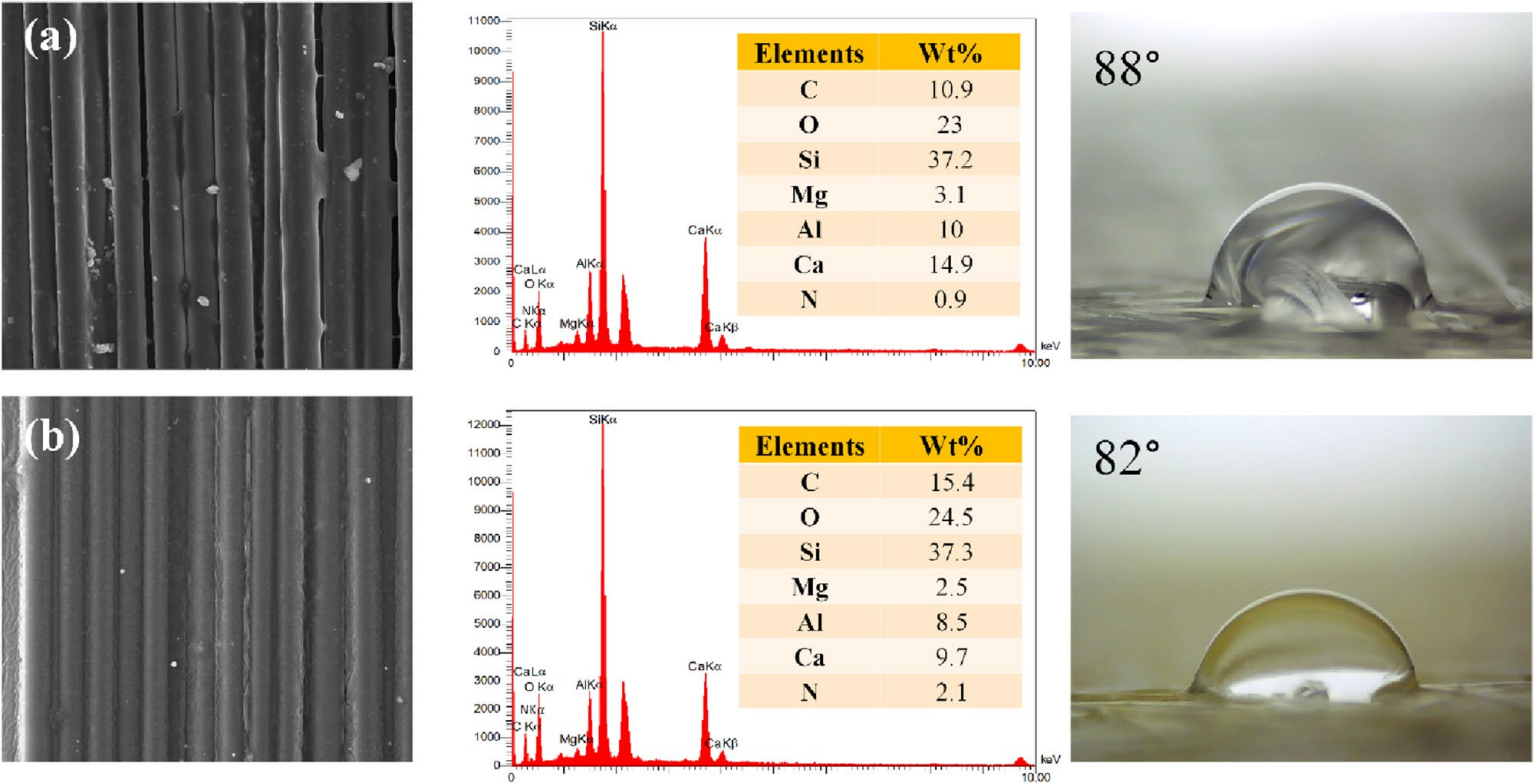
FESEM images, EDS analysis, and contact angle mesurements of (**a**) GF and (**b**) MGF.

Figure 3 illustrates the surface morphology and elemental composition of GF and MGF, which was evaluated by FE-SEM, EDS, and contact angle analysis. The FE-SEM image (shown in Fig. 3a) demonstrated that the GF is fibrous in shape, and the EDS confirmed that the strong peak belongs to silicon which showed that the main element of this sample is Si. Figure 3b revealed that the surface morphology of GF has not experienced any significant change after modification with APTES. The EDS analysis of GF illustrated that the weight ratio of O/Si is 0.62, which then rises to 0.66 for MGF. The composition (wt%) of carbon has increased from 10.9% in neat GF to 15.4% in MGF. The increase of the carbon, oxygen, and nitrogen content confirms the successful grafting of APTES on the surface of GF. The water contact angle results indicated a slight increase in the hydrophilicity of GF after modification which can be related to grafting APTES on the surface of GF and an increase in oxygen and nitrogen composition, confirmed by EDS results.

**Figure 4:**
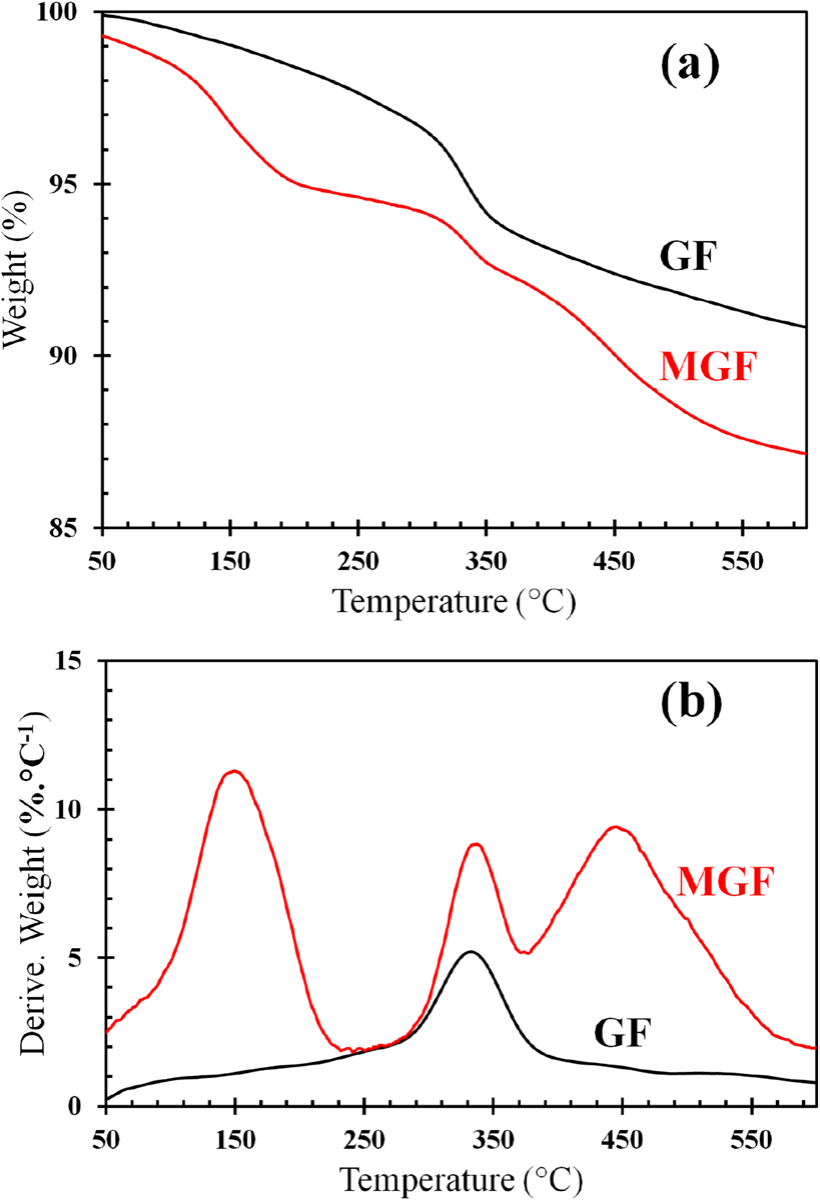
The TGA (**a**) and DTG (**b**) graphs of GF and MGF.

The thermogravimetric curves of GF and MGF are shown in Fig. 4. According to Fig. 4a, at the end of the test, the examined weight loss of MGF was about 4% higher than that of neat GF as the result of the APTES decomposition. The derivative thermogravimetric plots of two specimens are illustrated in Fig. 4b. The GF sample shows a single mass loss at around 350 °C attributed to the dehydroxylation. In addition to this weight loss, two other weight loss stages appeared for the MGF sample. The first one, centered at ca. 150 °C, is ascribed to the removal of water molecules due to the condensation of unreacted silanols groups. The second weight loss at 425 °C could be attributed to the decomposition of organic segments of APTES molecules^52,53^.

### Mechanical behavior of FRP

**Figure 5:**
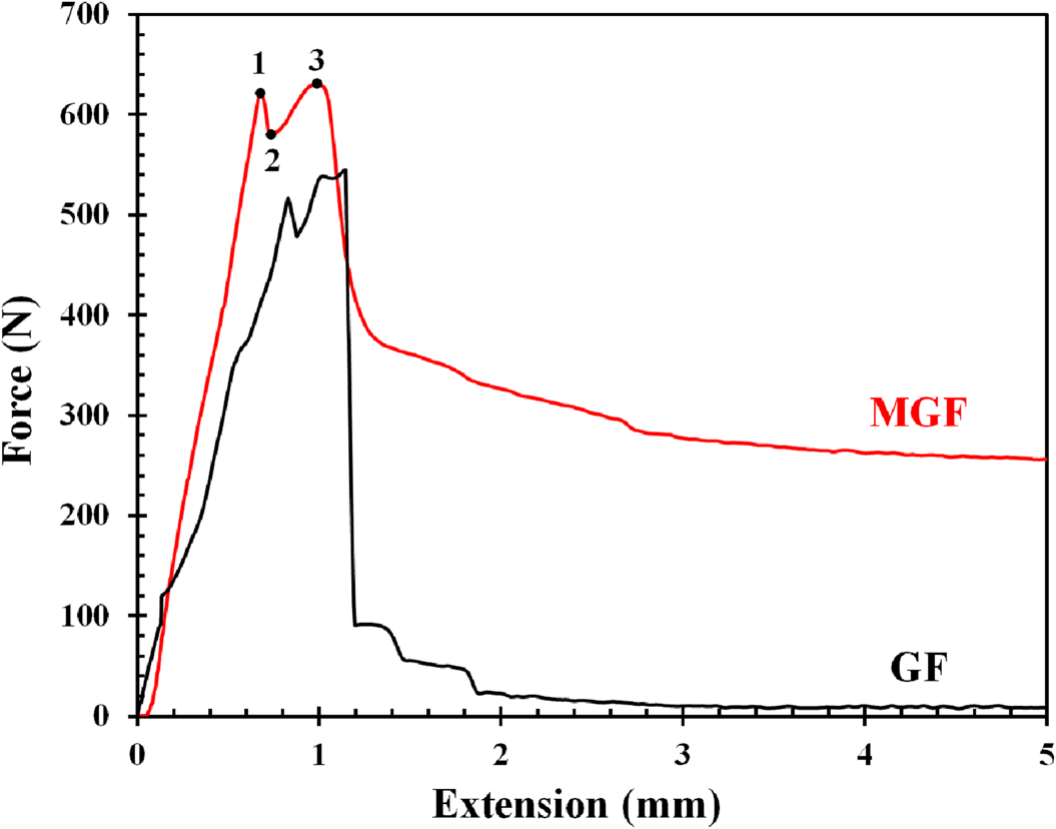
The tensile stress–strain test results of GF and MGF epoxy composites.

The tensile stress–strain diagram of the composite reinforced with MGF and the GF is shown in Fig. 5. According to the curves, the elastic deformation is followed by plastic deformation. Upper yield point (assigned as 1 in Fig. 5), lower yield point (2), ultimate tensile strength (number 3), and elongation were obtained from the curves. Due to the more significant amount of pores and cracks in GF, lower elongation and tensile strength were obtained compared to the MGF specimen. The highest force reported for MGF was 625 N, and for GF was around 550 N. There were oscillations shown in the curves of GF in the plastic region. The test indicated that MGF exhibited higher mechanical behavior and tensile strength in comparison to the GF sample.

**Figure 6:**
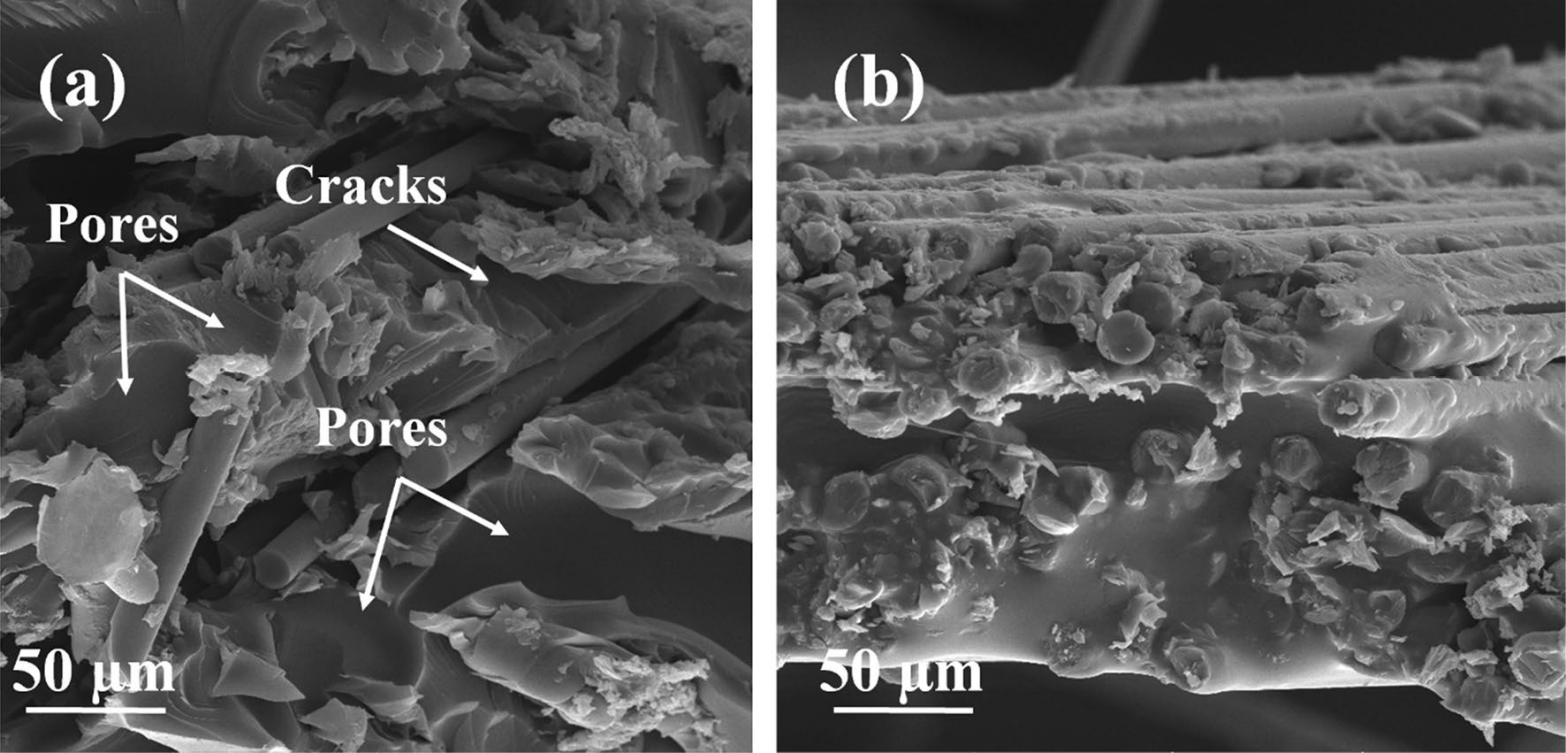
FESEM images of surface fracture of (**a**) GF and (**b**) MGF in epoxy composites.

Fracture surfaces of the composites after the tensile test examined by FE-SEM are shown in Fig. 6. At the magnification of 1000×; it is evident that cracks and the formation of pores found on the surface of GF (illustrated in Fig. 6a), which can be the initial site of fracture and failure. On the surface of the fracture specimens of MGF, there are no traces of cracks and voids. This also proved the better mechanical behavior of the MGF sample. The amino groups of APTES, which are grafted to MGF, can chemically react to epoxide groups of epoxy resin and make excellent compatibility to the matrix and avoid cracks and voids initiation.

### Cathodic delamination resistance of FRPs

**Figure 7:**
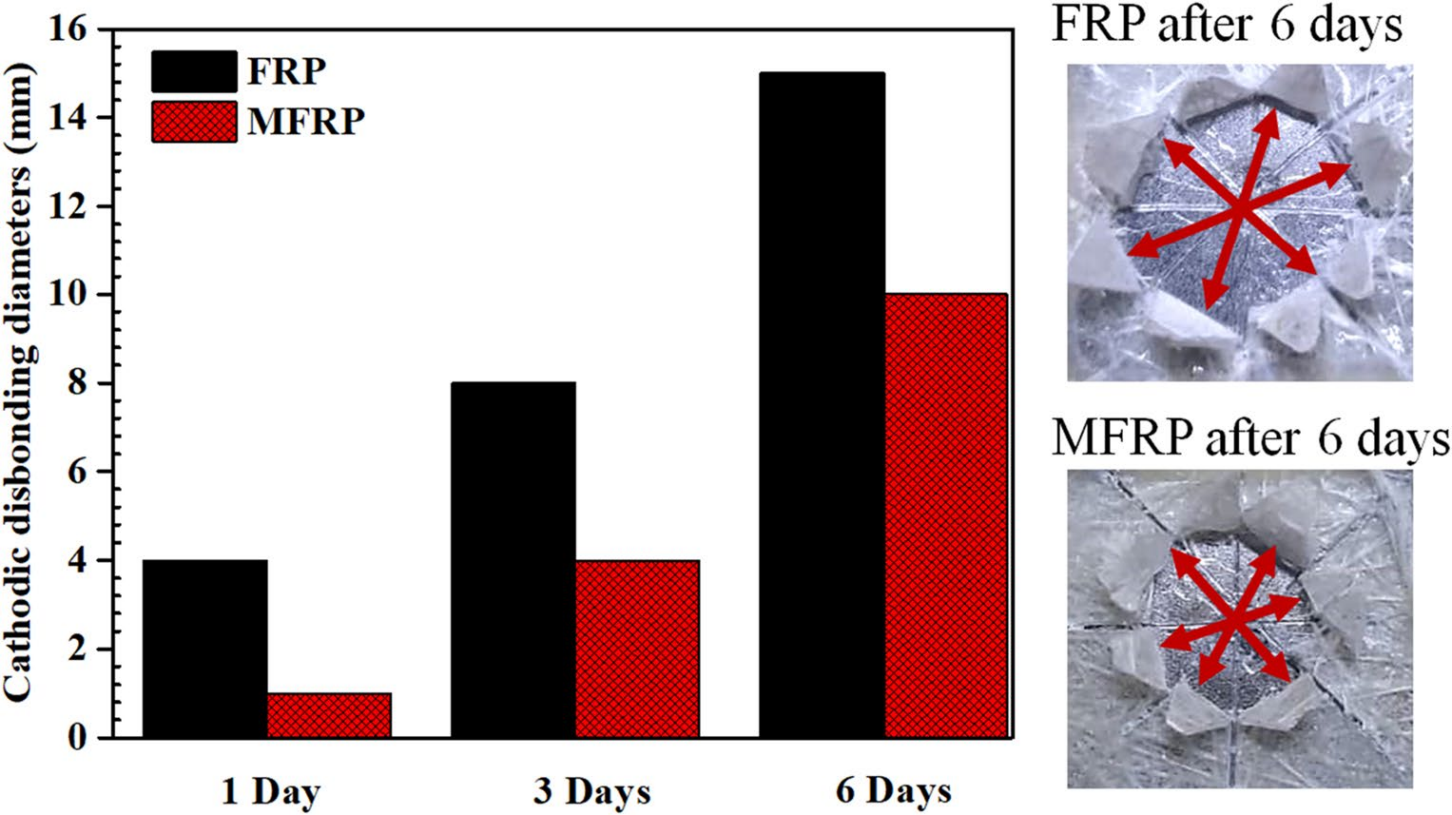
The visual delaminated area of FRP and MFRP during the cathodic delamination test.

The cathodic disbonding test was accomplished on the FRP and MFRP samples to investigate the adhesion of the coatings when the substrate was under cathodic protection. This method exerted the potential of − 1.5 V (vs SCE) to a mild steel substrate. The delaminated area of coatings was studied, and the outcomes are depicted in Fig. 7. The MFRP samples showed significantly lower delamination area compared to FRP, which can be referred to as less cathodic reactions at the metal/coating substrate due to fewer defects and pores in the MFRP coating. The water adsorbed in the coating during the time caused the cathodic reactions to take place beneath the coating and at the artificial hole. The cathodic reactions are shown in Eqs. (1) and (2). Due to releasing of hydroxyl ions, the pH at the interface was increased and caused the hydrolysis and destruction of coating/substrate bonding. The defects and pores in coatings cause the penetration of more water to the interface of coating/steel, enhancing the cathodic reaction, pH, and delamination:

1 $$2{H}_{2}O+ {O}_{2}+4e\to 4({OH}^{-})$$

2 $$2{H}_{2}O+ 2e\to {H}_{2}+ 2({OH}^{-})$$

**Figure 8:**
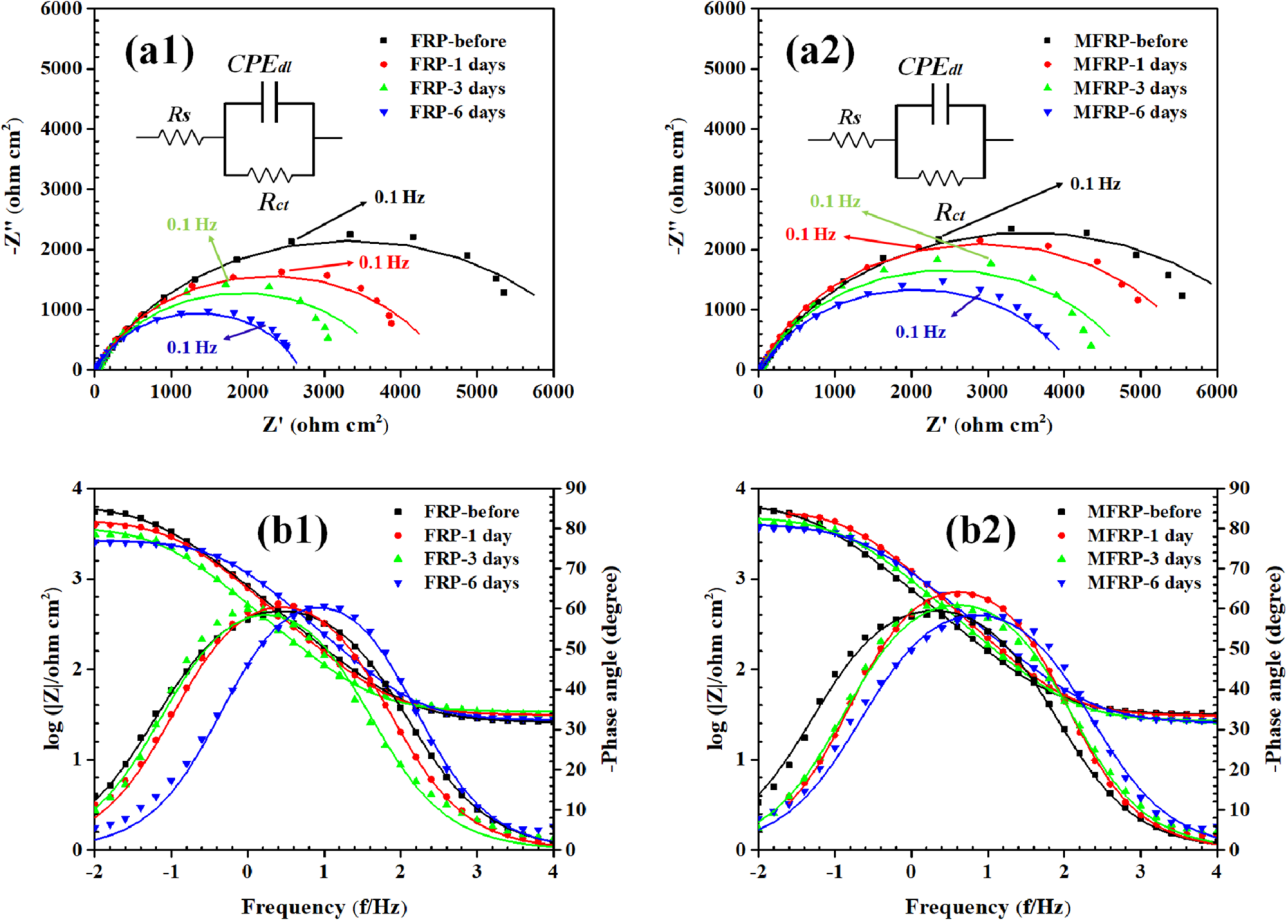
Nyquist plots of (**a1**) GF, (**a2**) MGF and Bod plots of (**b1**) GF, (**b2**) MGF from EIS measurements (symbols) and fitted data (line) during 6 days of exposure to the cathodic delamination condition.

**Figure 9:**
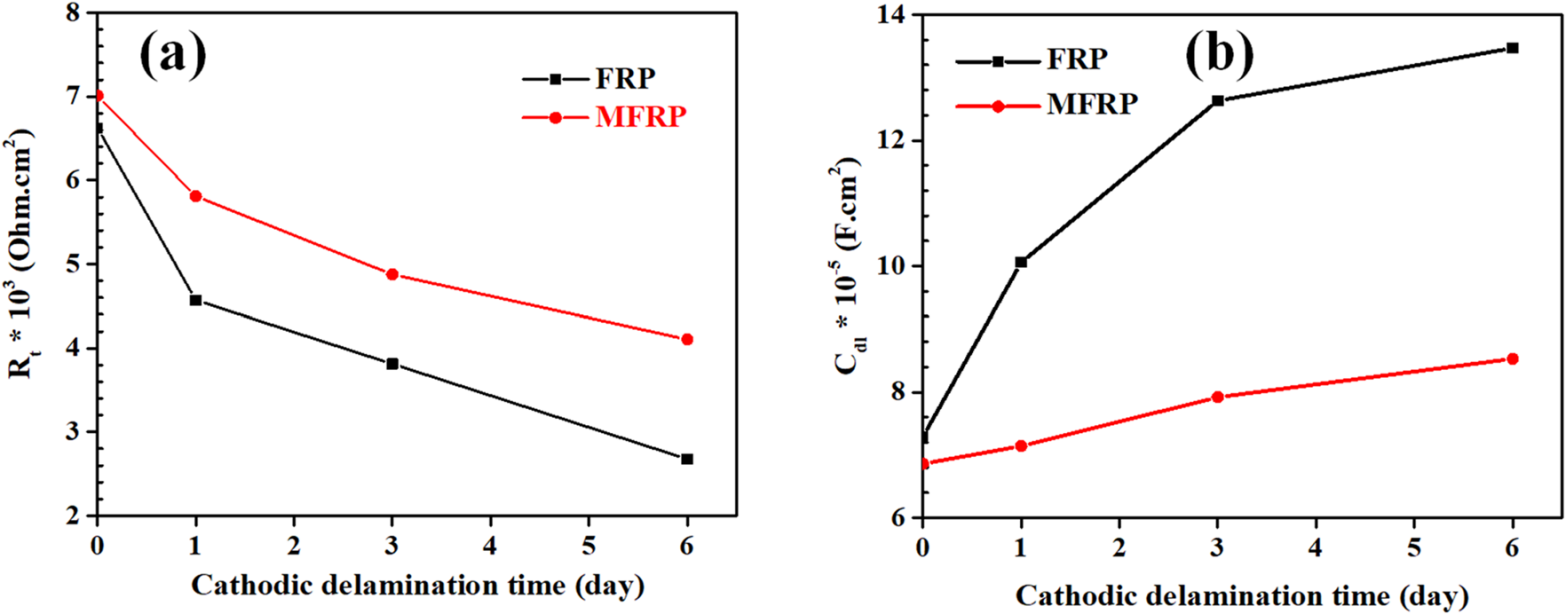
The FRP and MFRP’s (**a**) total resistance (R_t_) and (**b**) double layer capacitance during 6 days of exposure to the cathodic delamination condition.

Due to the high thickness of FRP coatings (approximately 0.5 mm), the delamination area of samples was further examined by EIS examination. The Nyquist and Bode diagrams of EIS results were depicted in Fig. 8a,b, respectively. From the Bode diagram, it can be seen that one relaxation time was attained, indicating the electrochemical reaction is under charge transfer control. The experimental results were simulated by a simple one-relaxation time-based electrical equivalent circuit (EEC) (Fig. 8), in which *R*_s_, *R*_ct_, and CPE_dl_ are, respectively, solution resistance, charge transfer resistance, and double layer constant phase element. The fitting results are provided in Table 1. The capacitance of the double layer is an appropriate parameter providing a measure of the delaminated surface, which was computed by Eq. (3)^54^:

3 $${C}_{dl}= {Y}_{0dl}^{\left(\frac{1}{{n}_{dl}}\right)} \times ({\frac{{R}_{s}+ {R}_{ct}}{{R}_{s} \times {R}_{ct}})}^{\frac{(1-{n}_{dl})}{{n}_{dl}}}$$

where Y_0dl_ and n_dl_ are double-layer admittance and inhomogeneity-related constant of double layer, respectively. The total resistance (*R*_t_ = *R*_ct_ + *R*_s_) and *C*_dl_ of specimens before and during the cathodic delamination test were depicted in Fig. 9a,b, respectively. Before the test, the artificial hole of specimens was the same, which confirmed the similar *R*_t_ and *C*_dl_ of samples before the delamination test. The increment of *C*_dl_ and reduction of *R*_t_ result in spreading the delamination area over time. After 6 days of the cathodic delamination test, the *C*_dl_ and *R*_t_ ratios of MFRP/FRP coatings were 0.6 and 1.6, respectively, illustrating the high impact of MGF in epoxy coating's delamination resistance. The findings align with the information presented in the Bode diagrams (Fig. 9b). In the Bode plots, the time constant of the samples was associated with both the charge transfer resistance and the constant phase element (CPE). These elements exhibited a shift to higher frequencies as the immersion time increased, indicating an increase in the time constant (*R*_t_ × *C*_dl_) values^55,56^.

**Table 1:** Fitted EEC parameters of FRP and MFRP samples during the cathodic delamination test immersed in 3.5 wt% NaCl solution.

Sample	Cathodic delamination time	*R*_s_ (ohm cm^2^)	*R*_ct_ (kohm cm^2^)	CPE_dl_	
				*Y*_0_ (ohm^−1^ cm^−2^ s^n^)	*n*
FRP	Before test	27 ± 2	6.96 ± 0.7	3.25 × 10^–4^	0.78 ± 0.03
	1 day	25 ± 1	6.6 ± 0.61	3 × 10^–4^	0.8 ± 0.02
	3 days	31 ± 3	4.54 ± 0.34	2.9 × 10^–4^	0.8 ± 0.06
	6 days	33 ± 2	3.78 ± 0.26	4.5 × 10^–4^	0.75 ± 0.04
MFRP	Before test	29 ± 1	7.03 ± 0.45	3.32 × 10^–4^	0.76 ± 0.05
	1 day	31 ± 4	6.98 ± 0.39	3.2 × 10^–4^	0.75 ± 0.04
	3 days	30 ± 4	5.78 ± 0.31	1.7 × 10^–4^	0.79 ± 0.06
	6 days	26 ± 2	4.85 ± 0.13	2.3 × 10^–4^	0.76 ± 0.06

**Figure 10:**
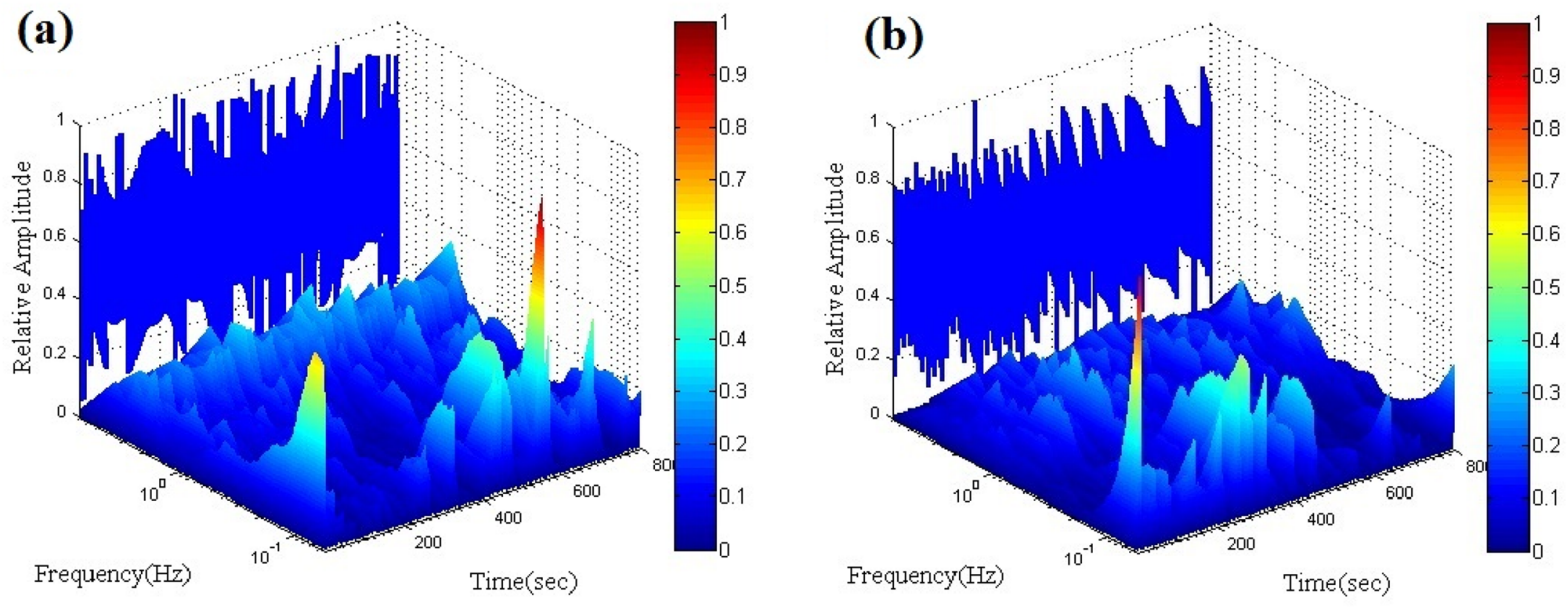
Electrochemical current noise signals from the FRP (**a**) and MFRP (**b**) samples after 6 days of exposure to the cathodic delamination condition.

Current noise transients derived from the wavelet transform are provided in Fig. 10. According to the figure, both FRP and MFRP shows the distribution of transients in the whole frequency range; however, MFRP shows a bit higher contribution of transients at higher frequency region, which might be ascribed to the less electrochemically active sites on the MFRP sample. The total energy of the current noise signal was 647 and 809 μA^2^ for the MFRP and FRP, revealing a reduction in the active site on the mild steel due to the less delamination of the coating from the surface.

**Figure 11:**
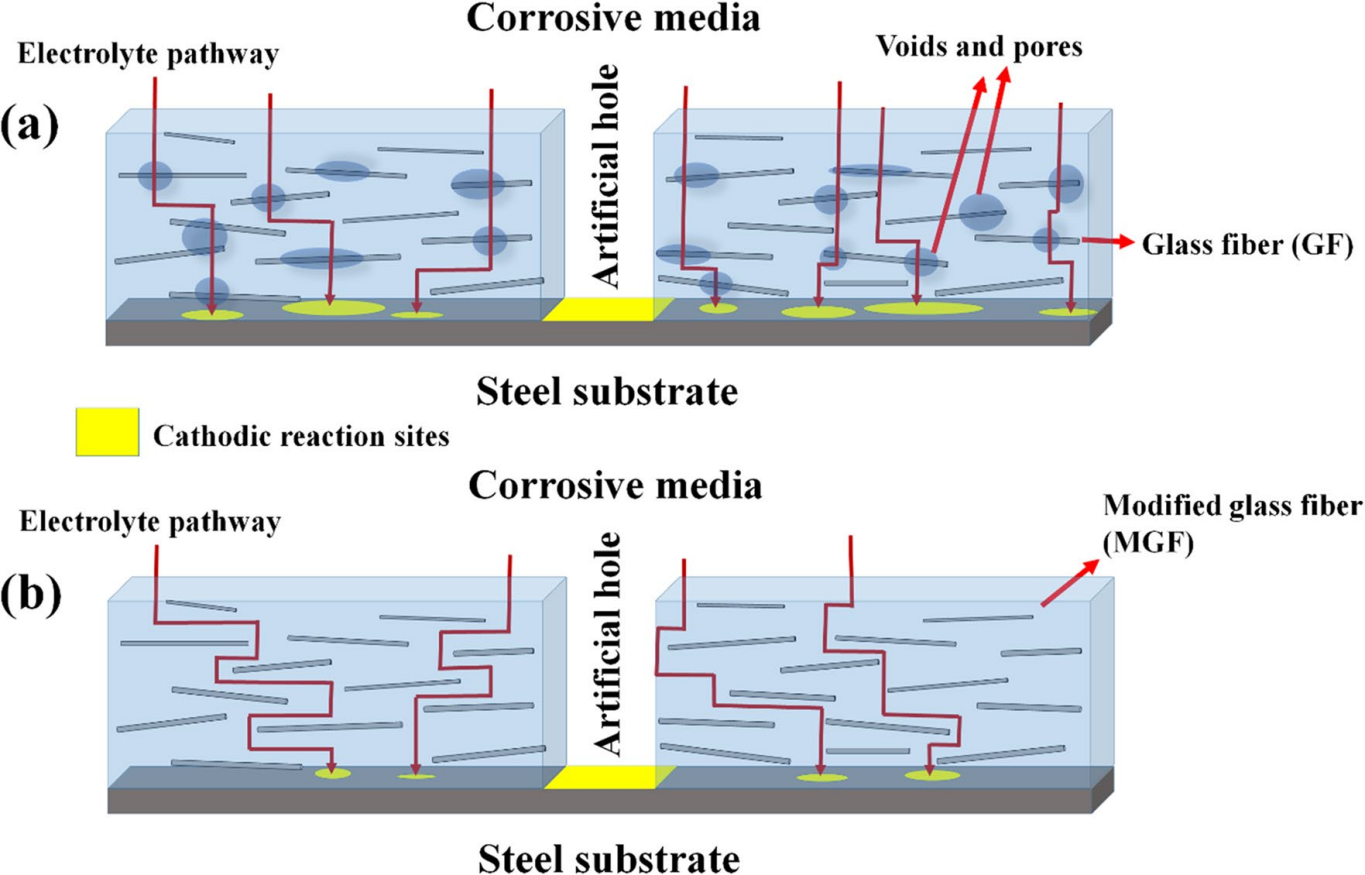
Schematic of water penetration and cathodic reaction sites in (**a**) FRP and (**b**) MFRP during cathodic delamination test.

A schematic presentation of the effect of modification of GF on the cathodic delamination resistance is provided in Fig. 11. Presence of defects between unmodified GF and epoxy matrix provides a path for the water to penetrate in the FRP. In the case of MFRP, the MGF is wholly embedded in the epoxy matrix, and there is almost no defect at the MGF–epoxy interface. Water molecules take a longer path to reach the metal-coating interface. The lower water penetration in MFRP compared to FRP reduces the cathodic reaction rates on the substrate surface, provoking less coating disbondment.

### Dry and wet adhesion strength measurements

**Figure 12:**
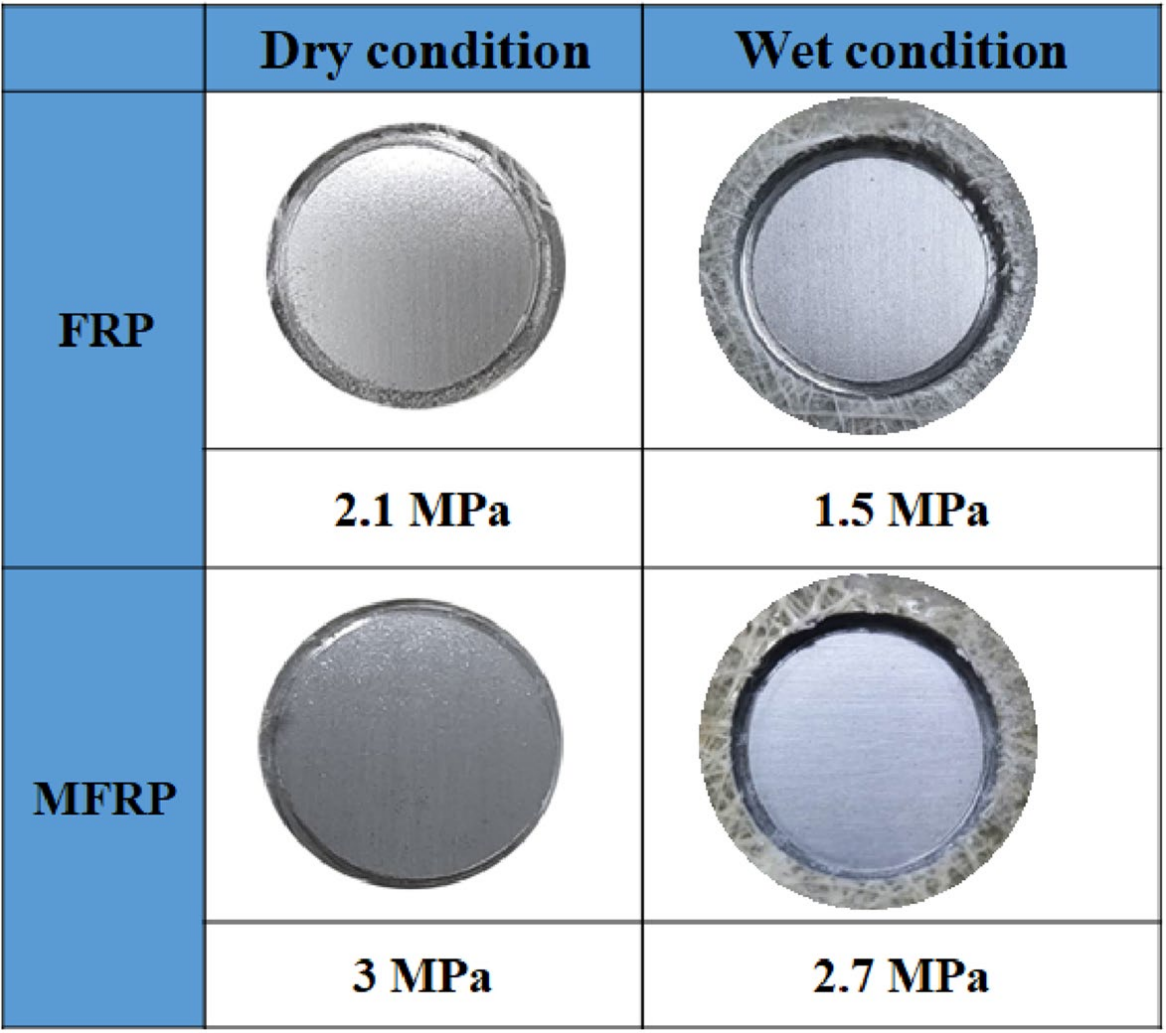
Pull-off adhesion results under dry and wet condition for the FRP and MFRP samples.

The pull-off measurement results are given in Fig. 12. In dry conditions, it is clear that the MFRP (3 MPa) had more adhesion strength compared to the unmodified specimen (2.1 MPa). The samples that underwent a 720-h salt spray test were considered in their wet state. The adhesion results in the wet state revealed significantly higher adhesion strength for MFRP (2.7 MPa) compared to FRP (1.5 MPa). The adhesion loss percentage was calculated by Eq. (4):

4 $$adhesion\, loss= \left(\frac{D-W}{D}\right) \times 100$$

where *D* and *W* are referred to the samples' strength of adhesion in dry and wet states. A lower adhesion loss was detected in the MFRP sample (10%) compared to the FRP (28%), reflecting improved durability of adhesion of the coatings upon surface modification of GFs.

## Conclusion

- The GFs were successfully modified by APTES through the condensation reaction of the hydroxyl groups of GFs and silanol groups of APTES.
- A cathodic delamination test evaluated the corrosion protection and compatibility of modified GFs. The visual cathodic delamination showed that the delamination area of the composite decreased by 34% after modification.
- The EIS results indicated a ca. 40% increase in double-layer capacitance and a ca. 40% decrease in the charge transfer resistance after 6 days of the cathodic delamination test, showing higher delamination of FRP compared to MFRP.
- The EN results also revealed a decrease in the current noise signal energy (ca. 20%) upon treatment of GFs, confirming the EIS results.
- The mechanical results of FRP and MFRP composites exhibited higher mechanical behavior and tensile strength upon treatment of GFs.
- According to the results of this work, the modification of GFs can effectively improve the FRP's cathodic resistance and mechanical strength.

## Data Availability

All data generated or analyzed during this study are included in this published article.
